# Supplementary material for: Deregulated DNA ADP-ribosylation impairs telomere replication
Source: Nat Struct Mol Biol. Author manuscript; Available in PMC 2025 May 7. (PMC11102865; doi:10.1038/s41594-024-01279-6)
Supplement: Antibodies Used [file NIHMS1992609-supplement-Antibodies_Used.pdf]

Supplementary Table 1

Antibodies used in IF:

Antibody	Source	Identifier	Dilution
FLAG (D6W5B)	Cell Signaling Technology	Cat. #14793S	1:1000
FLAG (M2)	Millipore	Cat. #F1804	1:1000
TRF1	Abcam	Cat. #ab10579	1:1000
TRF2	Novus	Cat. #NB110-57130	1:1000
RPA2	Abcam	Cat. #ab2175<br>Lot #GR3386072-1	1:1000
Poly/Mono-ADP-ribose	Cell Signaling Technology	Cat. #83732, Lot #5	1:1000
Mono-ADP-ribose	Biorad	Cat. #HCA354<br>Lot #162452	1:1000
Mono-ADP-ribose	Biorad	Cat. #HCA355<br>Lot #158442	1:1000
Cas9	Cell Signaling Technology	Cat. #14697<br>Lot #8	1:1000
GFP	Abcam	Cat. #ab6556	1:1000
Alexa Fluor 594 goat anti-rabbit	Thermo Fisher	Cat. #A11012<br>Lot #2433881	1:1000
Alexa Fluor 594 goat anti-mouse	Thermo Fisher	Cat. #A11032<br>Lot #2527968	1:1000
Alexa Fluor 488 goat anti-mouse	Thermo Fisher	Cat. #A11001<br>Lot #2659299	1:1000
Alexa Fluor 488 goat anti-rabbit	Thermo Fisher	Cat. #A32731<br>Lot #XD343356	1:1000
Alexa Fluor 647 goat anti-mouse	Thermo Fisher	Cat. #A21244<br>Lot #2527970	1:1000

Antibodies used in dot-blot:

Antibody	Source	Identifier	Dilution
Pan-ADP-ribose	Millipore	Cat. #MABE1016	1:1000
Poly/Mono-ADP Ribose	Cell Signaling	Cat. #83732S, Lot #5	1:1000
Mono-ADP-ribose	Biorad	Cat. #HCA354	1:1000
Auto anti-dsDNA	University of Iowa, Developmental Studies Hybridoma Bank	Cat. #AB_10805293	1:200
Auto anti-ssDNA	University of Iowa, Developmental Studies Hybridoma Bank	Cat. #AB_10805144	1:200

Antibodies used in western:

Antibody	Source	Identifier	Dilution
pRPA32 (S33)	Bethyl	Cat. #A300-246A, Lot #12	1:1000
pRPA32 (S4/S8)	Bethyl	Cat. #A300-245A, Lot #9	1:1000
pChk1 (S317)	Cell Signaling Technology	Cat. #2344S, Lot #12	1:1000
Chk1	Cell Signaling Technology	Cat. #37010S, Lot #1	1:1000

γH2AX	Cell Signaling Technology	Cat. #2577S Lot #12	1:1000
H2AX	Cell Signaling Technology	Cat. #2595S Lot #8	1:1000
GFP	Miltenyi Biotech	Cat. #130-091-833<br>Lot #5240106116	1:5000
PARG	Cell Signaling Technology	Cat. #66564<br>Lot #1	1:1000
PARP1	Active Motif	Cat. #61639<br>Lot #22922002	1:1000
PARP2	Active Motif	Cat. #39044<br>Lot #23822008	1:1000
TARG1	Proteintech	Cat. #25249-1-AP<br>Lot #00106148	1:1000
Pan-ADP-ribose	Millipore	Cat. #MABE1016	1:1000
Poly/Mono-ADP Ribose	Cell Signaling Technology	Cat. #83732S<br>Lot #5	1:1000
Cyclin E2	Cell Signaling Technology	Cat. #4132S<br>Lot #3	1:1000
HPF1	Novus	Cat. #NBP1-93973<br>Lot #000004509	1:1000
ARH3	Santa Cruz	Cat. #sc-374162<br>Lot #G0919	1:500
Tubulin	Sigma-Aldrich	Cat. #T6557	1:5000
Poly-ADP-ribose (10H)	Enzo	Cat. #ALX-804-220-R100	1:1000
FEN1	Fortis Life Sciences	Cat. #A300-256A, Lot #2	1:1000
DNA Ligase 1	Fortis Life Sciences	Cat. #A301-136A	1:1000
POT1	Novus	Cat. #NB500-176	1:1000
Myc	Thermo Fisher	Cat. #MA1-21316	1:1000
