# Supplementary material for: Deregulated DNA ADP-ribosylation impairs telomere replication
Source: Nat Struct Mol Biol. 2024 May 7;31(5):791–800. doi: 10.1038/s41594-024-01279-6 (PMC11102865; doi:10.1038/s41594-024-01279-6)

Related to ED 2A.

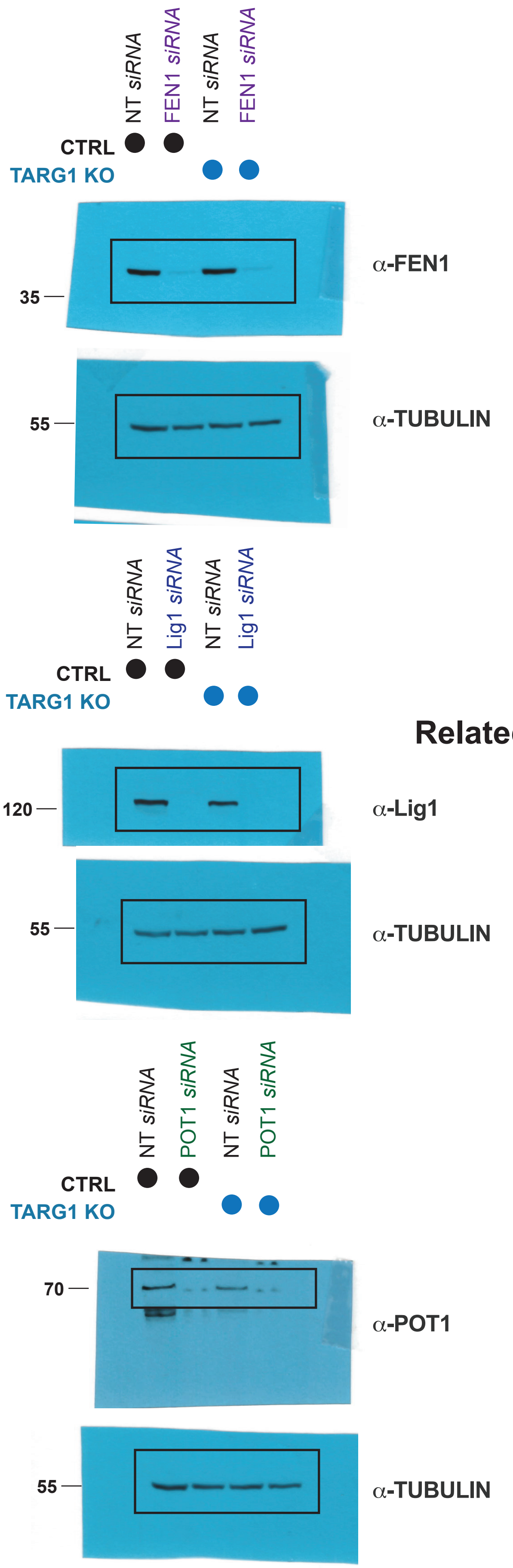

Related to ED 2F.

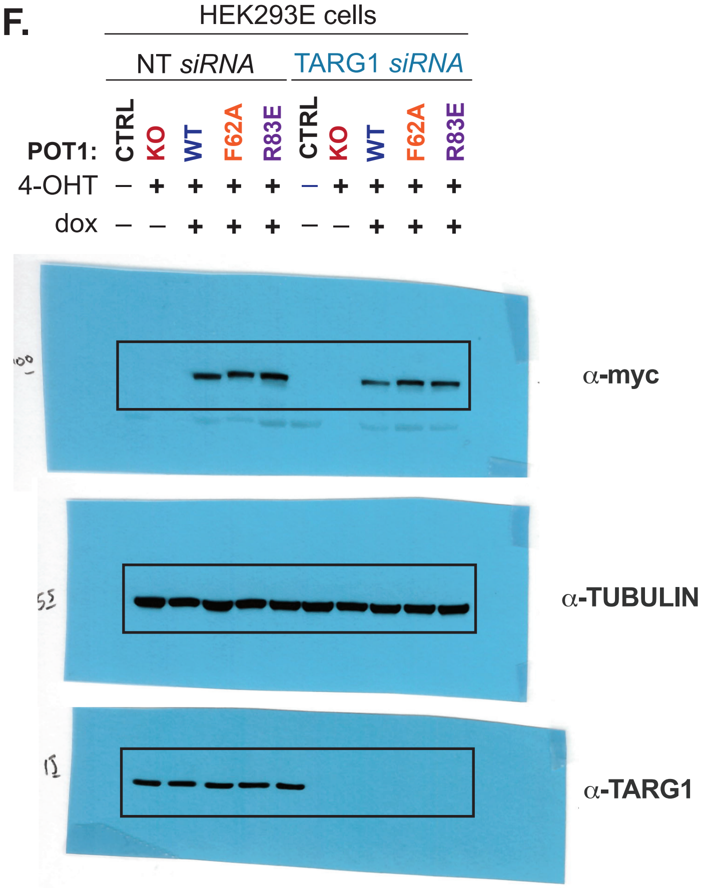

Related to ED 2D.

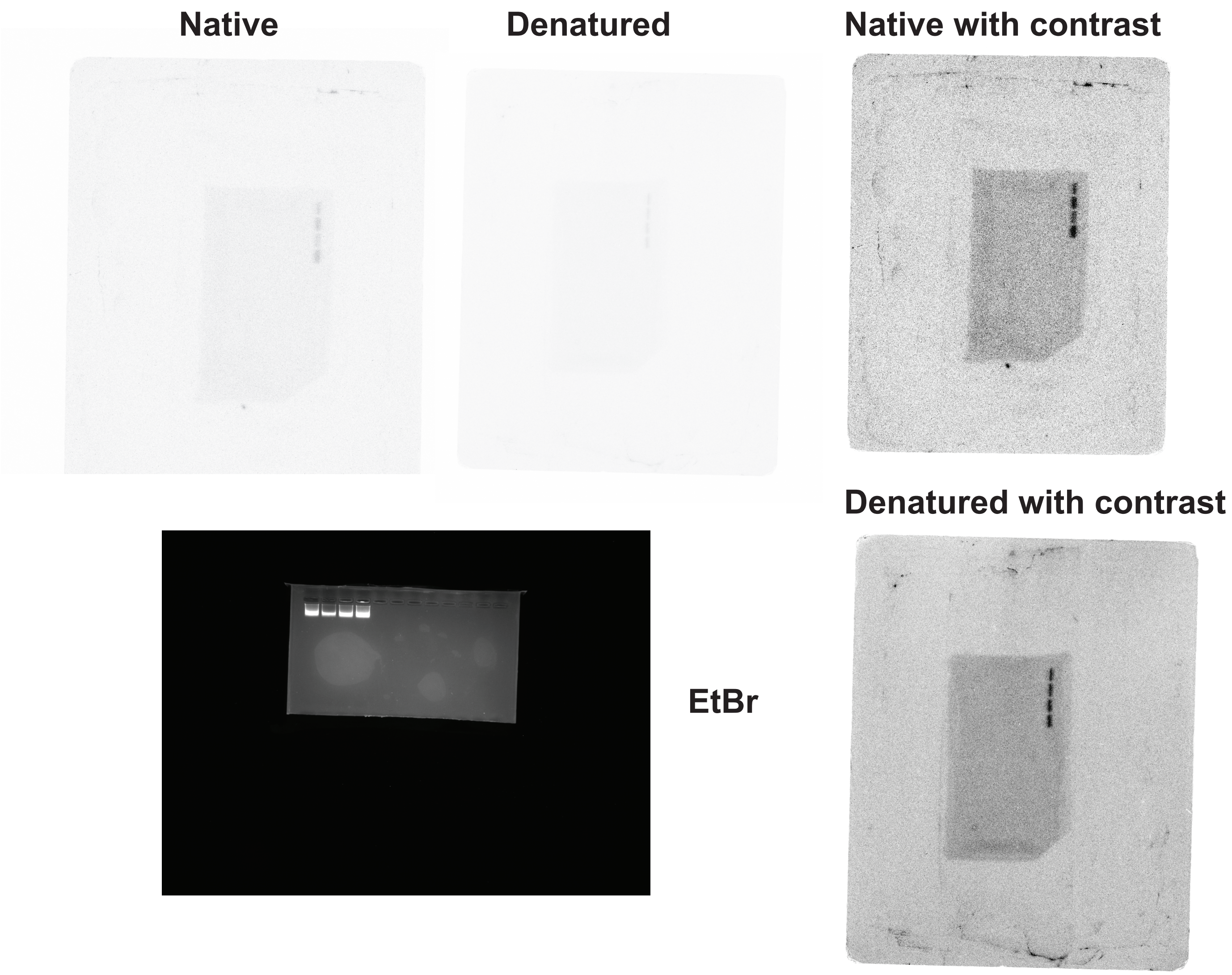

Supplement: Supplementary file 2 [file 41594_2024_1279_MOESM13_ESM.pdf]
