# Supplementary material for: Deregulated DNA ADP-ribosylation impairs telomere replication
Source: Nat Struct Mol Biol. 2024 May 7;31(5):791–800. doi: 10.1038/s41594-024-01279-6 (PMC11102865; doi:10.1038/s41594-024-01279-6)
Supplement: Supplementary file 4 [file 41594_2024_1279_MOESM1_ESM.pdf]

nature portfolio

Corresponding author(s): Roderick J. O'Sullivan

Last updated by author(s): Mar 11, 2024

Reporting Summary

Nature Portfolio wishes to improve the reproducibility of the work that we publish. This form provides structure for consistency and transparency in reporting. For further information on Nature Portfolio policies, see our Editorial Policies and the Editorial Policy Checklist.

Statistics

For all statistical analyses, confirm that the following items are present in the figure legend, table legend, main text, or Methods section.

n/a	Confirmed	
☐	☒	The exact sample size (*n*) for each experimental group/condition, given as a discrete number and unit of measurement
☐	☒	A statement on whether measurements were taken from distinct samples or whether the same sample was measured repeatedly
☐	☒	The statistical test(s) used AND whether they are one- or two-sided<br>*Only common tests should be described solely by name; describe more complex techniques in the Methods section.*
☒	☐	A description of all covariates tested
☐	☒	A description of any assumptions or corrections, such as tests of normality and adjustment for multiple comparisons
☐	☒	A full description of the statistical parameters including central tendency (e.g. means) or other basic estimates (e.g. regression coefficient) AND variation (e.g. standard deviation) or associated estimates of uncertainty (e.g. confidence intervals)
☐	☒	For null hypothesis testing, the test statistic (e.g. F, t, r) with confidence intervals, effect sizes, degrees of freedom and P value noted<br>*Give P values as exact values whenever suitable.*
☒	☐	For Bayesian analysis, information on the choice of priors and Markov chain Monte Carlo settings
☒	☐	For hierarchical and complex designs, identification of the appropriate level for tests and full reporting of outcomes
☒	☐	Estimates of effect sizes (e.g. Cohen's *d*, Pearson's *r*), indicating how they were calculated

Our web collection on statistics for biologists contains articles on many of the points above.

Software and code

Policy information about availability of computer code

Data collection	Nikon 90I Microscope, NIS Elements
Data analysis	GraphPad Prism (10.0.0), ImageJ2 (Version: 2.14.0/1.54f), and Microsoft Excel (16.66), ComDet v.0.5.3 plugin for ImageJ, Zen

For manuscripts utilizing custom algorithms or software that are central to the research but not yet described in published literature, software must be made available to editors and reviewers. We strongly encourage code deposition in a community repository (e.g. GitHub). See the Nature Portfolio guidelines for submitting code & software for further information.

Data

Policy information about availability of data

All manuscripts must include a data availability statement. This statement should provide the following information, where applicable:

- Accession codes, unique identifiers, or web links for publicly available datasets
- A description of any restrictions on data availability
- For clinical datasets or third party data, please ensure that the statement adheres to our policy

All data are available in the main, extended data, and supplementary data figures. Original data are deposited at Figshare doi:10.6084/m9.figshare.25103732 or available from the corresponding author upon request.

Research involving human participants, their data, or biological material

Policy information about studies with human participants or human data. See also policy information about sex, gender (identity/presentation), and sexual orientation and race, ethnicity and racism.

Reporting on sex and gender	N/A
Reporting on race, ethnicity, or other socially relevant groupings	N/A
Population characteristics	N/A
Recruitment	N/A
Ethics oversight	N/A

Note that full information on the approval of the study protocol must also be provided in the manuscript.

Field-specific reporting

Please select the one below that is the best fit for your research. If you are not sure, read the appropriate sections before making your selection.

☒ Life sciences ☐ Behavioural & social sciences ☐ Ecological, evolutionary & environmental sciences

For a reference copy of the document with all sections, see nature.com/documents/nr-reporting-summary-flat.pdf

Life sciences study design

All studies must disclose on these points even when the disclosure is negative.

Sample size	No statistical method was used to predetermine sample size. Sample size was determined based on previous experiments. For metaphases, n=20-30 metaphases over 3 independent experiments for telomere fragility and TSCEs.
Data exclusions	No data were excluded from the analyses.
Replication	The number of biologically independent experiments performed is indicated in the legends. Experiments are representative of at least n=3 independent experiments unless indicated. All attempt at replication were found to be successful. Several experiments independently conducted by different researchers.Samples prepared in Pittsburgh were shipped to Oxford and independently assayed (Extended Data 1B) - this provided additional confirmation of TARG1 hydrolytic activity against DNA-ADPr.<br>Cell lines were shipped to dr PIckette annd Mins laboratories where experiments on ADPr at telomeres were corroborated
Randomization	Randomization was done when possible. Nuclei and metaphase imaging for all treatments were taken randomly.
Blinding	Investigators were not blinded except for metaphases experiments where investigators were blinded to the samples during imaging and analysis. All samples were treated identically for all experiments.

Reporting for specific materials, systems and methods

We require information from authors about some types of materials, experimental systems and methods used in many studies. Here, indicate whether each material, system or method listed is relevant to your study. If you are not sure if a list item applies to your research, read the appropriate section before selecting a response.

Materials & experimental systems

n/a	Involved in the study	
☐	☒	Antibodies
☐	☒	Eukaryotic cell lines
☒	☐	Palaeontology and archaeology
☒	☐	Animals and other organisms
☒	☐	Clinical data
☒	☐	Dual use research of concern
☒	☐	Plants

Methods

n/a	Involved in the study	
☒	☐	ChIP-seq
☒	☐	Flow cytometry
☒	☐	MRI-based neuroimaging

Antibodies

Antibodies used	Immunofluorescence: FLAG (D6W5B) (#14793S, Cell Signaling Technology, 1:1000); FLAG (M2) (#F1804, Millipore, 1:1000); TRF1 (#ab10579, Abcam, 1:1000); TRF2 (#NB110-57130, Novus, 1:1000); RPA2 (#ab2175 Lot #GR3386072-1, Abcam, 1:1000), Poly/Mono-ADP-ribose (#83732, Lot #5, Cell Signaling Technology, 1:1000); Mono-ADP-ribose (#HCA354 Lot #162452, Biorad, 1:1000); Mono-ADP-ribose (#HCA355 Lot #158442, Biorad, 1:1000); Cas9 (Cat. #14697 Lot #8, Cell Signaling Technology, 1:1000); GFP #ab6556, Abcam, 1:1000); Alexa Fluor 594 goat anti-rabbit (#A11012 Lot #2433881, Thermo Fisher, 1:1000); Alexa Fluor 594 goat anti-mouse. (#A11032 Lot #2527968, Thermo Fisher, 1:1000); Alexa Fluor 488 goat anti-mouse (#A11001 Lot #2659299, Thermo Fisher, 1:1000); Alexa Fluor 488 goat anti-rabbit. (#A32731 Lot #XD343356, Thermo Fisher, 1:1000); Alexa Fluor 647 goat anti-mouse. (#A21244 Lot #2527970, Thermo Fisher, 1:1000).<br>Dot blot: Pan-ADP-ribose (#MABE1016, Millipore, 1:1000); Poly/Mono-ADP-ribose (#83732, Lot #5, Cell Signaling Technology, 1:1000); Mono-ADP-ribose (#HCA354 Lot #162452, Biorad, 1:1000); Auto anti-dsDNA (#AB_10805293, University of Iowa, Developmental Studies Hybridoma Bank, 1:200); Auto anti-ssDNA #AB_10805144, University of Iowa, Developmental Studies Hybridoma Bank, 1:200).<br>Western blot: pRPA32 (S33) (#A300-246A, Lot #12, Bethyl, 1:1000); pRPA32 (S4/S8) (#A300-245A, Lot #9, Bethyl, 1:1000); pChk1 (S317) (#2344S, Lot #12, Cell Signaling Technology, 1:1000); Chk1 (#37010S, Lot #1, Cell Signaling Technology, 1:1000); gH2AX (#2577S Lot #12, Cell Signaling Technology, 1:1000); H2AX (#2595S Lot #8, Cell Signaling Technology, 1:1000); GFP (#130-091-833 Lot #5240106116, Miltenyi Biotech, 1:5000); PARG (#66564 Lot #1, Cell Signaling Technology, 1:1000); PARP1 (#61639 Lot #22922002, Active Motif, 1:1000); PARP2 (#39044 Lot #23822008, Active Motif, 1:1000); TARG1 (#25249-1-AP Lot #00106148, Proteintech, 1:1000); Pan-ADP-ribose (#MABE1016, Millipore, 1:1000); Poly/Mono-ADP-ribose (#83732, Lot #5, Cell Signaling Technology, 1:1000); Cyclin E2 (#4132S Lot #3, Cell Signaling Technology, 1:1000); HPF1 (#NBP1-93973 Lot #000004509, Novus, 1:1000); ARH3 (#sc-374162 Lot #G0919, Santa Cruz, 1:500); Tubulin (#T6557, Sigma-Aldrich, 1:5000); Poly-ADP-ribose (10H) (#ALX-804-220-R100, Enzo, 1:1000); FEN1 (#A300-256A, Lot #2, Fortis Life Sciences, 1:1000); DNA Ligase 1 (#A301-136A, Fortis Life Sciences, 1:1000); POT1 (#NB500-176, Novus, 1:1000); Myc (#MA1-21316, Thermo Fisher, 1:1000).
Validation	PAR westerns were validated by sensitivity to PARP inhibitor. TARG1, PARP1 antibody validated by knockout of corresponding protein. siRNA depletion of corresponding targets proteins was used in validation of other antibodies. where possible, previously validated antibodies were obtained from commercial vendors. Antibodies were validated by the suppliers companies for reactivity against the human proteins.

Eukaryotic cell lines

Policy information about cell lines and Sex and Gender in Research

Cell line source(s)	U2OS (HTB-96) and IMR90 (CCL-96) cells were obtained from ATCC. Control and TARG1 KO U2OS and HeLa cells were generated and obtained from Ivan Ahel (Tromans-Coia C, Sanchi A, Moeller GK, Timinszky G, Lopes M, Ahel I, TARG1 protects against toxic DNA ADP-ribosylation, Nucleic Acids Research (2021)). Hek293E cell lines were obtained from Jayakrishna Nandakumar (Tesmer VM, Brenner KA, Nandakumar J. Human POT1 protects the telomeric ds-ss DNA junction by capping the 5' end of the chromosome. Science. (2023)).
Authentication	Cell lines validated by STR authentication at ATCC.
Mycoplasma contamination	Routinely tested negative using MycoAlert Kit (Lonza). Cells routinely treated my plasmocin prophylactic reagent. Incubators cleaned by high temp sanitization quarterly.
Commonly misidentified lines<br>(See ICLAC register)	HeLa, HEK293E

Plants

Seed stocks	N/A
Novel plant genotypes	N/A
Authentication	N/A
