# Supplementary material for: Deregulated DNA ADP-ribosylation impairs telomere replication
Source: Nat Struct Mol Biol. 2024 May 7;31(5):791–800. doi: 10.1038/s41594-024-01279-6 (PMC11102865; doi:10.1038/s41594-024-01279-6)

Related to 1A.

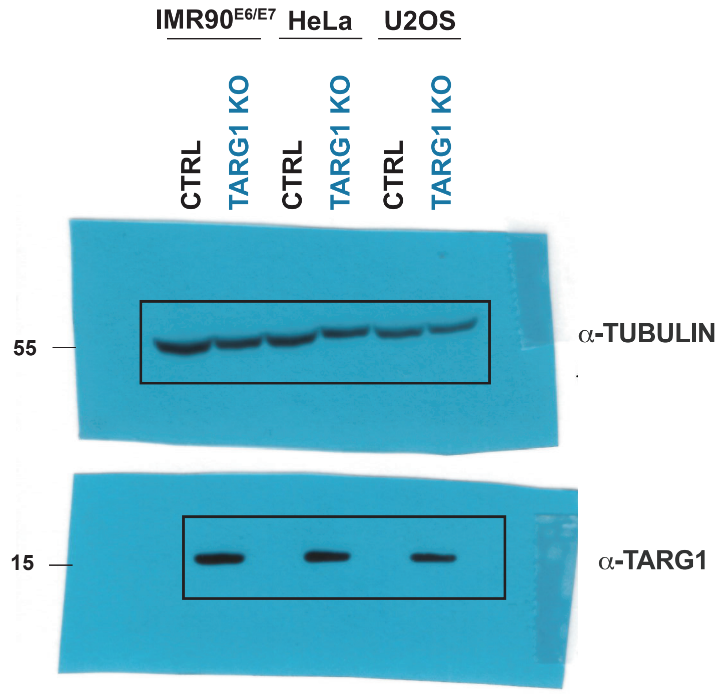

Related to 1B.

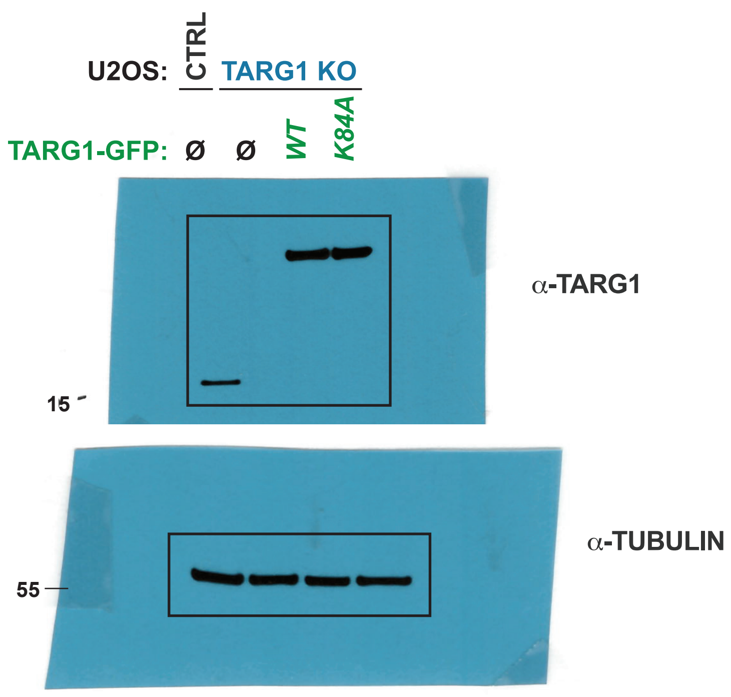

Related to 1C.

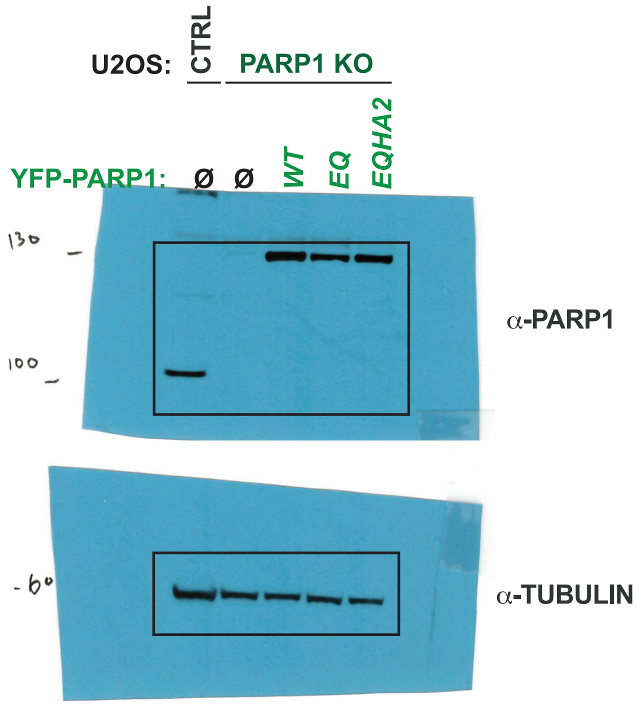

Related to 1E.

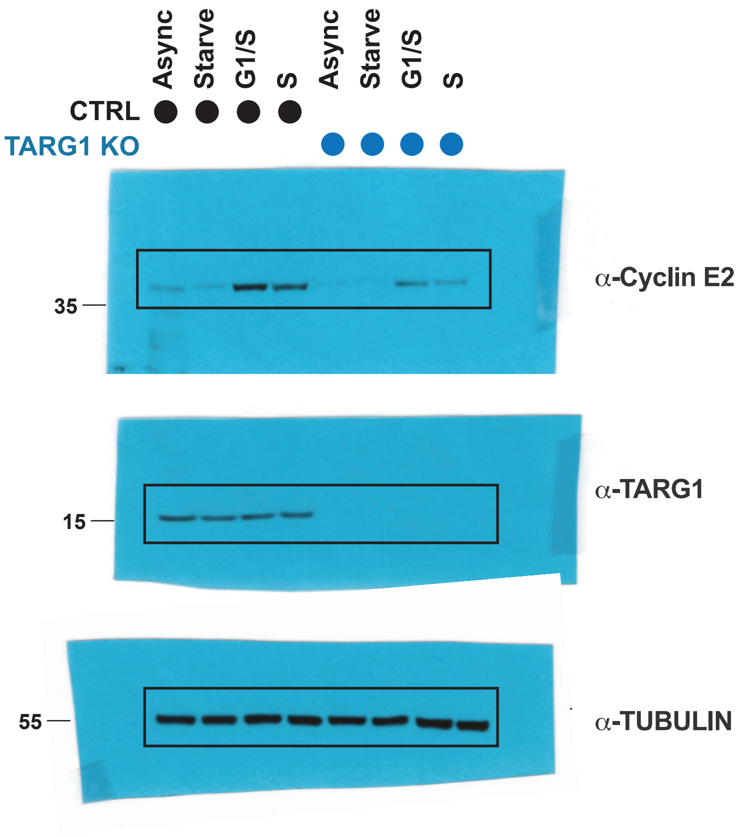

Supplement: Supplementary file 5 [file 41594_2024_1279_MOESM4_ESM.pdf]
