# Supplementary material for: Deregulated DNA ADP-ribosylation impairs telomere replication
Source: Nat Struct Mol Biol. 2024 May 7;31(5):791–800. doi: 10.1038/s41594-024-01279-6 (PMC11102865; doi:10.1038/s41594-024-01279-6)

Related to 2D.

Native Denatured EtBr

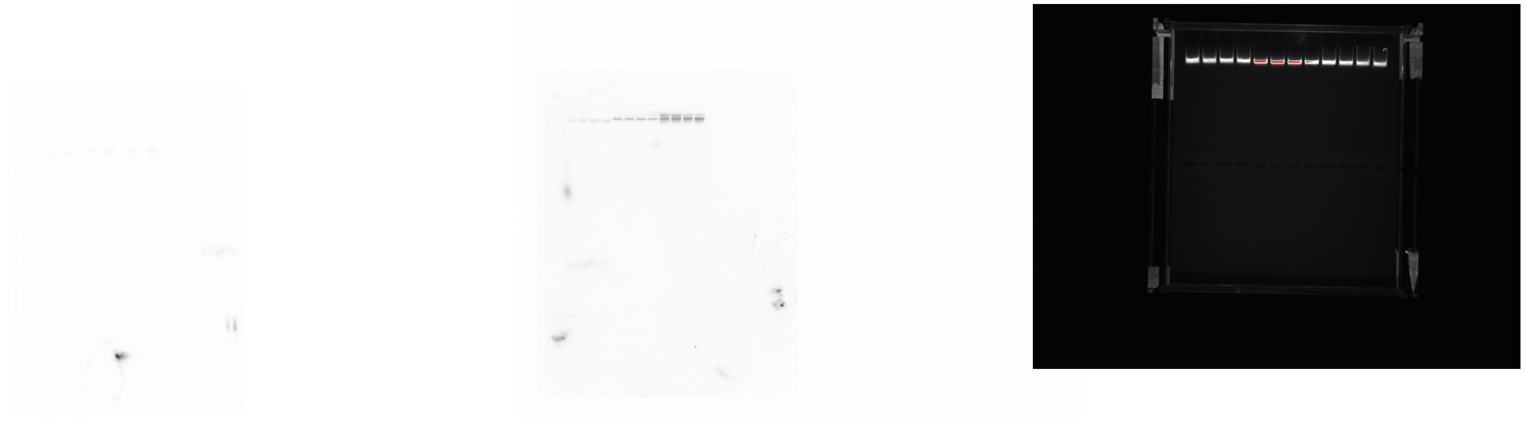

Related to 2D with contast.

Native Denatured

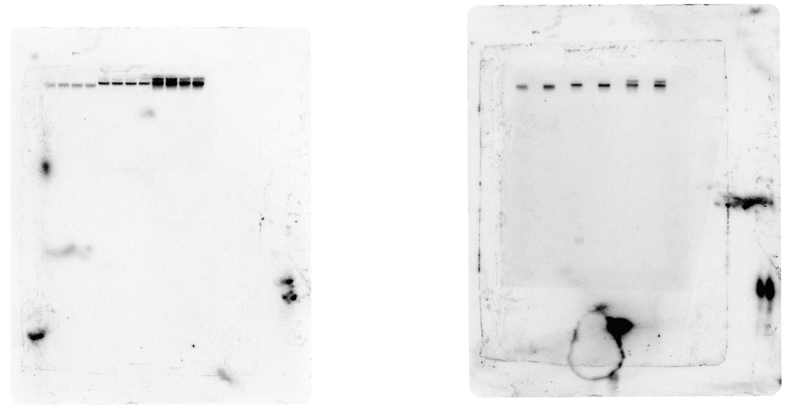

Related to 2F.

Native Denatured EtBr

Hela

U2OS
IMR90

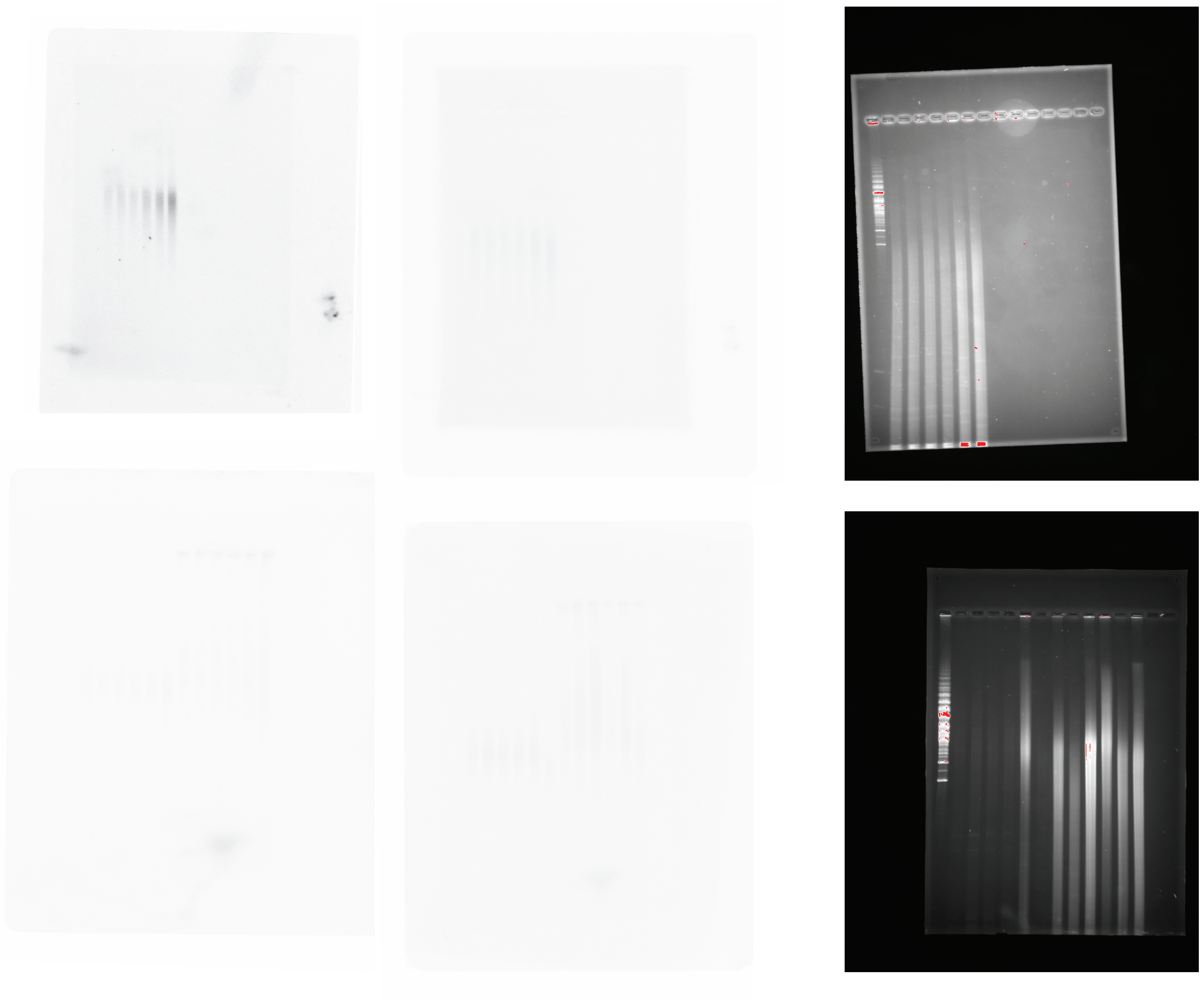

Related to 2F with contrast.

Native Denatured

Hela

U2OS
IMR90

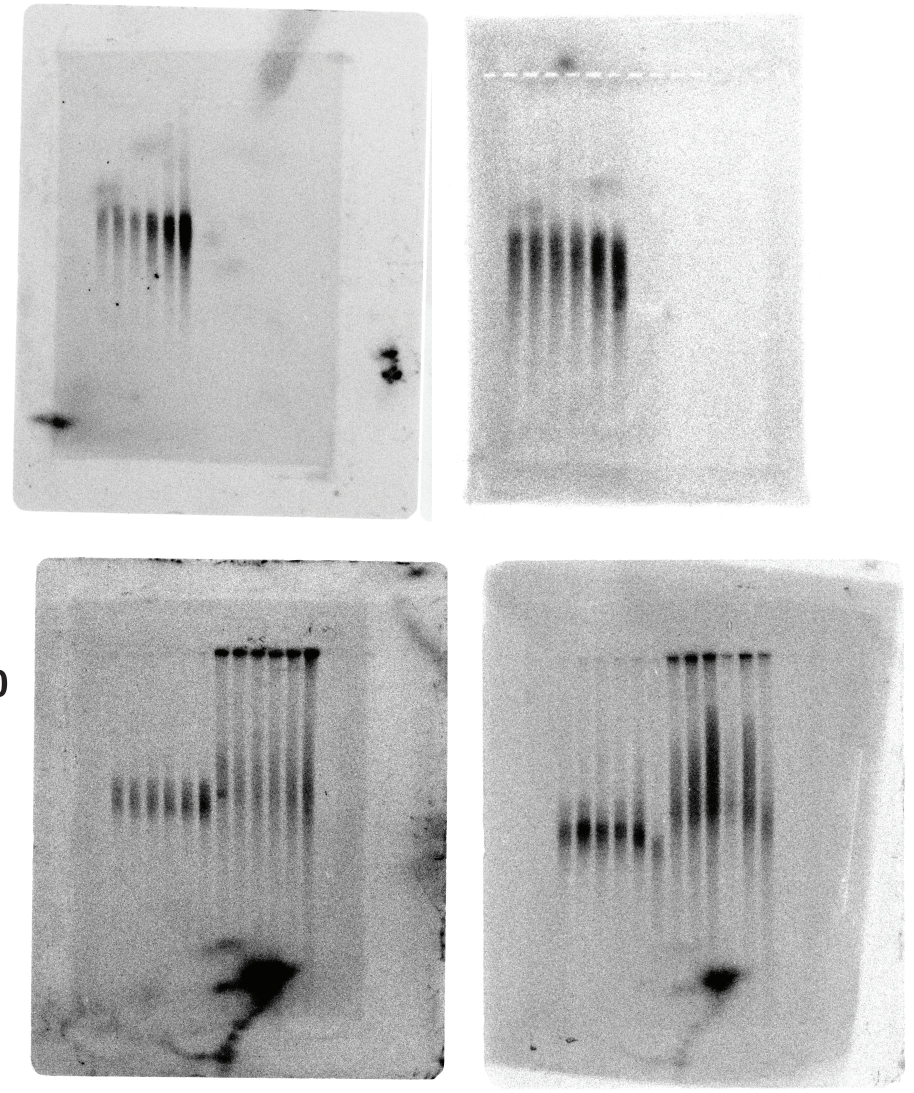

Supplement: Supplementary file 6 [file 41594_2024_1279_MOESM6_ESM.pdf]
